# Determinants of physical activity during cancer treatment: a longitudinal exploration of psycho-cognitive variables and physician counseling

**DOI:** 10.1007/s10865-023-00458-y

**Published:** 2023-11-28

**Authors:** Alexander Haussmann, Nadine Ungar, Angeliki Tsiouris, Laura I. Schmidt, Jana Müller, Jost von Hardenberg, Joachim Wiskemann, Karen Steindorf, Monika Sieverding

**Affiliations:** 1 Division of Physical Activity, Prevention and Cancer, German Cancer Research Center (DKFZ) and National Center for Tumor Diseases (NCT) Heidelberg, Im Neuenheimer Feld 581, 69120 Heidelberg, Germany; 2 Institute of Psychology, Heidelberg University, Hauptstraße 47-51, 69117 Heidelberg, Germany; 3 Faculty of Healthcare and Nursing, Catholic University of Applied Sciences Mainz, Saarstraße 3, 55122 Mainz, Germany; 4 Working Group Exercise Oncology, Division of Medical Oncology, National Center for Tumor Diseases (NCT) Heidelberg and Heidelberg University Hospital, Im Neuenheimer Feld 460, 69120 Heidelberg, Germany; 5 Department of Psychosomatic Medicine and Psychotherapy, University Medical Center Mainz, Johannes Gutenberg University Mainz, Untere Zahlbacher Straße 8, 55131 Mainz, Germany; 6 Department of Urology and Urological Surgery, Medical Faculty Mannheim, University Medical Centre Mannheim (UMM), University of Heidelberg, Theodor-Kutzer-Ufer 1-3, 68167 Mannheim, Germany

**Keywords:** Oncology, Physical activity, Supportive care, Counseling, Self-efficacy, Intention

## Abstract

Individuals with cancer are recommended to engage in regular physical activity (PA) even during cancer therapy. The aim of this study was to explore how patient-reported physician PA counseling influences their PA intention and behavior in addition to psycho-cognitive determinants derived from the theory of planned behavior (TPB). A longitudinal study during cancer treatment was conducted among N = 115 patients with breast, prostate, or colorectal cancer (*M*_age_ = 58.0, *SD* = 11.5; 55.7% female). The median time since diagnosis was 2 months, and 19.1% were diagnosed with metastases. Participants provided information on PA counseling by their physicians and on psycho-cognitive variables of the TPB at three measurement points. Additionally, they wore accelerometers for seven days at baseline and three months later. Nearly half of participants (48%) reported basic PA counseling and 30% reported in-depth PA counseling. Patients in poorer health and with lower education reported significantly less in-depth counseling. In addition to patient self-efficacy in performing PA, only in-depth physician PA counseling, but not basic physician counseling, predicted intention for PA four weeks later. Patients’ PA three months after baseline was predicted by patients’ PA at baseline and their intention for PA. Overall, the PA level at baseline was identified as the most important predictor of PA three months later. Nevertheless, physicians seem to have the ability to increase their cancer patients' intention for PA by in-depth counseling.

**Supplementary Information:**

The online version contains supplementary material available at 10.1007/s10865-023-00458-y.

## Introduction

In the past decades, a vast number of scientific publications have highlighted the benefits of physical activity (PA) for individuals living with cancer. The state of research on exercise and cancer was reviewed in a roundtable of international experts in 2018, organized by the American College of Sports Medicine (ACSM). The consortium found substantial evidence that sufficient PA increases survival in individuals with breast, prostate, and colorectal cancer (Patel et al., 2019). A recent meta-analysis of randomized controlled exercise trials in cancer patients and survivors found a 26% relative reduction in mortality risk in favor of exercise interventions (Morishita et al., 2020). The ACSM roundtable further underlined robust associations for the ameliorative effects of PA on eight common health-related outcomes of cancer or cancer therapies (e.g., cancer-related fatigue or chemotherapy-induced peripheral neuropathy) (Campbell et al., 2019). This scientific evidence led to the recommendation by the ACSM that individuals with cancer should engage in 150–300 min of moderate-to-vigorous PA per week (in addition to bi-weekly resistance training), also during cancer therapy.

However, most individuals with cancer are not sufficiently physically active, with only 30–50% meeting guidelines for aerobic exercise according to self-reports (Coletta et al., 2019; Crawford et al., 2016; Eng et al., 2018; Ottenbacher et al., 2015; Steindorf et al., 2019; Yan et al., 2018) and less than 15% according to accelerometer-derived activity data (McDonald et al., 2019; Smith et al., 2019; Thraen-Borowski et al., 2017). An important reason for unfavorable PA behaviors of cancer survivors seems to lie in a decrease of PA during chemotherapy or radiotherapy (Huy et al., 2012; Kenkhuis et al., 2021), or in the first weeks after cancer surgery (Smith et al., 2019; Zhou et al., 2022). Given the benefits of PA, it is worthwhile to elucidate which factors are essential for the development of PA levels during cancer treatment or post-surgery.

During cancer treatment, physicians have the potential to motivate individuals with cancer to engage in sufficient levels of PA (Schmitz et al., 2019). PA levels were found to be increased in those individuals with cancer who recalled a PA advice (Fisher et al., 2015; Tarasenko et al., 2017), as well as in study participants who received an oncologist-led PA recommendation in a randomized controlled trial (Jones et al., 2004). A systematic review by Brunet et al. (2020) pointed out that the intensity of PA counseling can make a difference in the effect on cancer patients’ PA levels, and that a simple recommendation is not necessarily sufficient to achieve behavior changes. A PA counseling based on the 5As framework goes beyond a recommendation for PA as it contains counseling steps defining basic counseling (*Assess* PA levels and give *Advice* on PA) but also in-depth counseling (*Agree* on PA goals, *Assist* in achieving them, and *Arrange* follow-up monitoring) (Estabrooks & Glasgow, 2006). A previous cross-sectional study with 1206 cancer survivors found evidence that only in-depth PA counseling (i.e., Agree, Assist, and/or Arrange), not basic PA counseling (i.e., Assess and/or Advise), was associated with higher levels of PA (Haussmann et al., 2021). Therefore, to gain more insights into the effects of PA counseling during cancer treatment, its impact on PA levels should be analyzed longitudinally and separately for basic and in-depth PA counseling.

In addition to PA counseling, psycho-cognitive variables play an important role for the engagement in PA by individuals with cancer (Hirschey et al., 2020). The Theory of Planned Behavior (TPB) (Ajzen, 1991) is a widely applied social cognitive theory to explain health behavior. In the TPB, the intention is the central determinant for explaining the health behavior. The intention, in turn, is influenced by self-efficacy, attitudes, and the perceived expectations by others (i.e., subjective norm). A meta-analysis of longitudinal observational studies indicated that assumptions of the TPB adequately reflect PA behavior of individuals with cancer (Hirschey et al., 2020). In the current study, we complemented the TPB with physician PA counseling as an additional determinant for PA intention and behavior. In addition, we considered it useful to assess cancer patients’ attitudes towards PA not only covering agreements with benefits of PA (Hunt-Shanks et al., 2006; Speed-Andrews et al., 2014; Trinh et al., 2012; Ungar et al., 2015). Benefits of PA for individuals with cancer are now widely acknowledged (activity paradigm); however, the attitude that cancer patients should rest during cancer therapy (rest paradigm) has not yet been completely overcome among health care professionals (Ungar et al., 2019) or among patients themselves (Haussmann et al., 2022).

The aims of this longitudinal observational study are to analyze (a) changes in PA behavior during cancer treatment or in the post-surgery phase, (b) perceived PA counseling provided by the attending physician taking into account basic and in-depth PA counseling, (c) differences in PA counseling according to different sociodemographic and disease-related characteristics, and (d) effects of physician counseling and psycho-cognitive variables on patients’ intention for PA and changes in PA levels.

## Methods

### Design

**Fig. 1:**
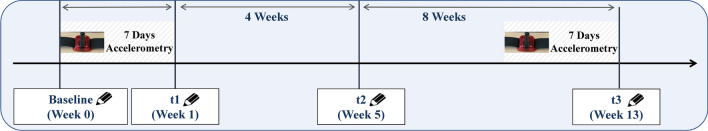
Study design

We conducted a longitudinal observational study over a period of three months. Participation in the study involved completion of four 10–15 min long paper–pencil questionnaires. The baseline assessment at t0 (hereafter referred to as baseline) was intended to occur as early as possible after cancer diagnosis, the other assessments followed one week later (t1), after 5 weeks (t2), and after 13 weeks (t3). Time points of assessments were not tied to treatment appointments with a physician. In addition, participants were asked to wear accelerometers for seven consecutive days at baseline (before t1) and immediately before t3 (cf. Figure 1).

The study received ethical approval by the ethics commission of the Faculty of Behavioral and Cultural Studies of Heidelberg University [AZ Siev 2015/1-1, AZ Siev 2016/1-2]. Informed consent was obtained from all participants prior to study participation.

The research concept was based on the TPB. Thus, psycho-cognitive determinants of the TPB at t1 as well as physician PA counseling before t2 were hypothesized to explain intention at t2, and intention, in turn, was expected to explain PA at t3. Physician PA counseling between t2 and t3 was hypothesized to moderate the effect of intention (t2) on PA (t3; cf. research concept in Online Resource 1).

### Participants and procedures

From April 2017 to February 2018, participants were recruited at the National Center for Tumor Diseases Heidelberg, at the Department of Urology and Urological Surgery of the University Medical Centre Mannheim, at the Radiooncology Department of the Heidelberg University Hospital, at the Clinic for Urology of the Heidelberg University Hospital, and six other clinics or practices. Potentially eligible study participants were identified based on information from the electronic clinical documentation system. Study staff approached potential participants in the day clinics or waiting rooms, informed them about the study and asked for their consent to participate in the study.

The study was also promoted through (online-)magazines and newsletters. Our goal was to include 60 evaluable patients with breast, colorectal or prostate cancer (thus, total N of 180) to potentially conduct subgroup analyses. Interested persons were further screened for inclusion criteria and received study material (questionnaires and accelerometer).

To be eligible for the study, participants had to (a) be between 18 and 80 years old, (b) receive the diagnosis of breast, colorectal, or prostate cancer within the past 24 months (including diagnosis of metastases or cancer recurrence), (c) be able to be physically active without assistive devices, and (d) be at the beginning of cancer therapy (including chemotherapy, radiotherapy, and anti-androgen therapy), or having received a surgery within the last three months. Beginning of therapy was dependent on the treatment type, defined as (a) the first two cycles of chemotherapy, (b) the first two weeks after the beginning of radiotherapy, or (c) six weeks after the beginning of anti-androgen-therapy. Patients who had undergone surgery were included after sufficient time for convalescence (six weeks post-surgery for breast cancer patients, eight weeks post-surgery for prostate and colorectal cancer patients). Time for convalescence was reduced in case the surgeon allowed PA earlier after surgery (for example due to minimal-invasive surgery methods). Participants were excluded if they received adjuvant chemotherapy before radiotherapy, if they had bone metastases, or if they simultaneously participated in an exercise intervention study. In general, inclusion criteria were chosen to recruit a sample of adult cancer patients being (potentially) physically and cognitively fit enough to engage in at least moderate unsupervised PA.

### Measures

#### Physical activity data collection and validation

PA was measured with the triaxial ActiGraph GT3X + accelerometer (ActiGraph, LLC, Pensacola, FL). Participants were asked to wear the device on an elastic belt around the waist for seven consecutive days in the waking hours, except for activities involving water. The data analysis was performed with ActiLife software (v6.11.6). Non-wear time was defined by an interval with a minimum length of 90 consecutive minutes of zero counts per minute (cpm). In addition, non-zero counts with a spike tolerance of two minutes interruptions were assigned as non-wear time if no activity counts were identified 30 min before and after these interruptions. To classify PA intensities, cpm were used as cut-offs according to Freedson et al. (1998): light PA (100 − 1951 cpm), moderate PA (1952 − 5724 cpm), and vigorous PA (> 5724 cpm). Moderate and vigorous activity were added to calculate moderate-to-vigorous PA. Accelerometer measurements were considered valid if the minimum wearing time was four days and the accelerometer was worn at least 10 h per day (Choi algorithm) (Choi et al., 2011).

Additionally, at baseline, we assessed self-reported PA before diagnosis and during last week using a modified version of the Godin–Shephard Leisure-Time Physical Activity Questionnaire (Godin, 2011) and calculated a mean score of minutes of moderate-to-vigorous PA per week. However, we used this self-reported PA data only for descriptive purposes (since subjective and objective measures of PA are often not highly correlated) and focused on the accelerometer-derived PA measure for the main analyses.

#### Physical activity counseling based on 5As framework (t1/t2/t3)

Participants were asked to indicate the counseling steps their physician provided on PA (since cancer diagnosis or the last questionnaire, respectively). In this regard, participants were asked to think of their current main attending physician or—if not present—their last treating physician. The assessed counseling steps followed the 5As framework (Estabrooks & Glasgow, 2006) (multiple answers possible): (1) ***A****ssess*: asked about current PA levels, (2) ***A****dvise*: provided recommendations on PA (on patient or physician initiative), (3) ***A****gree*: established PA goals or plans, (4) ***A****ssist*: helped achieve PA goals, and (5) ***A****rrange*: follow-ups to check if PA recommendations were met. Participants could additionally indicate that they did not receive any PA counseling. PA counseling was classified into no PA counseling, basic counseling (reported use of at least one of the counseling steps: *Assess* or *Advise* but no further counseling step), and in-depth counseling (reported use of at least one of the counseling steps: *Agree*, *Assist*, or *Arrange,* regardless of whether it was preceded by basic counseling).

#### Variables of the theory of planned behavior (t1/t2)

All variables of the TPB were developed based on the recommendations by Ajzen and Fishbein (Ajzen, 2002; Fishbein & Ajzen, 2011). Items were developed using a qualitative elicitation study including a quantitative pretest (Ungar et al., 2015) and were used in former studies (Haussmann et al., 2022; Ungar et al., 2016, 2019). Attitudes towards PA were assessed based on two dimensions according to previous cross-sectional studies from the same project (Haussmann et al., 2022; Ungar et al., 2019), i.e., activity paradigm (e.g., “physical activity improves my prognosis”) and rest paradigm (e.g., “I should conserve my energy for the actual therapy”). Answers on seven items of the activity paradigm and eight items of the rest paradigm had to be given on a 5-point Likert-scale (ranging from 1 = ‘completely disagree’ to 5 = ‘completely agree’). Cronbach’s alphas were α = 0.88 and α = 0.78 for the activity and the rest paradigm, respectively. Subjective norm was assessed with three items asking about perceived expectations by (1) partner/family, (2) physicians, and (3) nurses to engage in at least 150 min of moderate PA per week (e.g., “My partner or family thinks that I should spend at least 150 min per week of at least moderate PA”, Cronbach’s α = 0.87). Answers were given on a 7-point Likert-scale (from 1 = ‘completely disagree’ to 7 = ‘completely agree’). Self-efficacy in performing PA was assessed with two items (e.g., “I am sure I could manage to be at least moderately physically active for a minimum of 150 min per week”), also using a 7-point Likert-scale with a Cronbach’s alpha of α = 0.81. Behavioral intention for PA was assessed with one item asking for the intention to be physically active (“Please think about the next three months. Do you intend to regularly engage in at least 150 min of moderate PA per week?”; 7-point Likert-scale) and the second item asking for the subjective likelihood of pursuing this intention (as a percentage from 0 to 100). A mean value was calculated from these two items with a Cronbach’s alpha of α = 0.93 (Sieverding et al., 2010).

#### Personal information and disease-related variables (baseline)

Participants reported their personal information (i.e., sex, age, weight, height, education) and the following disease-related variables: cancer entity, date of cancer diagnosis, administered treatments, and, if applicable, diagnosis and date of metastases and cancer recurrence. If available, information on diagnosis, treatment, and metastases was validated using medical reports that study participants were asked to send along at the end of the study (done by 109 of 115 participants). Medical reports often specified dates by month only, so we summarized them at the month level for all participants. Eleven comorbidities were assessed in line with previous studies (e.g., Hoffman et al., 2005; Mohan & Schellhammer, 2011) that used an adapted version of the Charlson Comorbidity Index (Charlson et al., 1987) and an expert opinion by the investigators of the Prostate Cancer Outcomes Study (Potosky et al, 1999). In detail, chest pain was removed and we specified the item “arthritis” to “disease of the hip or knee joints or spine (arthritis or arthrosis)”.

Furthermore, a self-generated list (oriented towards an applied scale in a study among healthcare professionals (Haussmann et al., 2018a) asked participants about the presence of 15 common side effects of their cancer therapy (e.g., fatigue or neuropathy). Participants further assessed their general health status during the last week based on the Short-Form-Health-Survey-12 (SF-12) on a 7-point Likert Scale (Ware et al., 1996).

### Statistical analyses

Statistical analyses were performed using IBM SPSS version 27. A *p*-value < 0.05 was deemed statistically significant. Descriptive analyses were applied to determine the sociodemographic and medical characteristics of the study sample at baseline, their PA behavior at both accelerometer measurements, and their perceived physician PA counseling behavior at t1, t2, and t3.

Chi square tests were used to identify differences regarding the reported PA counseling between participants with different sociodemographic and disease-related characteristics. Differences regarding the proportions within a category of PA counseling were determined using z-tests with Bonferroni correction.

Longitudinal relationships based on the TPB explaining participants’ intention for PA (t2; secondary outcome) and accelerometer-derived moderate-to-vigorous PA (t3; primary outcome) were analyzed using two structural equation models (SEM) with Amos 27. Both SEM followed the structure of the TPB, but one model additionally included general PA counseling (sum of PA counseling at t1/t2, regardless if basic or in-depth PA counseling), the other specifically in-depth counseling (sum of in-depth PA counseling at t1 and t2). Subjective norm, self-efficacy, and intention were treated as latent variables, with their items considered as their respective indicators. For reasons of parsimony, the mean scores of the items of the activity and rest attitude scales were used to account for the sample size in this study relative to the number of variables (Shi et al., 2019); the 2-factorial structure of the attitude dimensions was presented elsewhere (Haussmann et al., 2022). For the same reason, no control variables were included in this model. We checked whether our data met the assumptions of structural equation modeling (normality of data based on Shapiro–Wilk test, bivariate normality between all variables using Quantile–Quantile (Q-Q) plots, missing data completely at random using Little’s Missing at Random Test, and linear relationships between variables using scatter plots). Due to the skewness of the intention variable (left skewed) and the moderate-to-vigorous PA variables at baseline and t3 (both right skewed) leading to a significant result in the Shapiro–Wilk test, they were transformed using natural logarithmic (ln) transformation. The other assumptions were met. Different indices were used to evaluate model fits (Hu & Bentler, 1999): Chi-square test, CMIN/DF (the ratio of chi-squared to degrees of freedom; ≤ 0.5 ≈ adequate; ≤ 0.3 ≈ good model fit), root mean square error of approximation (RMSEA; ≤ 0.08 ≈ adequate; ≤ 0.05 ≈ good model fit), as well as Comparative Fix Index (CFI), and Tucker-Lewis Index (TLI) (both: ≥ 0.90 ≈ adequate; ≥ 0.95 ≈ good model fit). Missing values were imputed in AMOS based on full information likelihood estimation.

We further tested the moderation of physician PA counseling (t3) in the effect of intention (t2) on moderate-to-vigorous PA (t3) as dependent variable. Hayes’ PROCESS macro for SPSS (version 4.1, Model 1) (Hayes, 2017), was applied to test for the simple moderation while no differentiation was made between basic and in-depth PA counseling (due to the low mention of the latter at t3). Exploratively, we examined whether this moderation may only or particularly apply for specific subgroups (Model 3 in Process). Correspondingly, 14 three-way moderation models were tested using all sociodemographic and disease-related variables (see Table 1) as well as treatment types as second moderator. Assumptions for moderation analyses (linear relationships between variables, uncorrelatedness of residuals, homoscedasticity, normality of residuals) were tested in advance. Although residuals were normally distributed according to the Shapiro–Wilk test (*p* = 0.113), visual inspections of the Q–Q plots showed substantial deviations from the normal distribution, especially in higher quantiles, so we opted for the more conservative bootstrapping procedure, which does not involve any assumptions about distributions of variables (Hayes, 2017). Confidence intervals (CI) with 95% accuracy were estimated for all moderation effects using 10,000-sample bootstrap procedures. Models were adjusted for sociodemographic/disease-related variables that were bivariately correlated with moderate-to-vigorous PA at t3 (i.e., moderate-to-vigorous PA at baseline, BMI (t2), metastasis, current chemotherapy (t3), sum of side effects (t3)), as well as for age and sex, and self-efficacy (t1).

**Table 1:** Sociodemographic and health-related characteristics at baseline and differences in reported physical activity counseling of the attending physician during study period

	Total		No PA Counseling^a^	Basic PA Counseling^b^	In-depth PA Counseling^c^	Test of differences^d^	
	N^e^	%	%	%	%	Χ^2^	*p*
Sex						1.53	.467
Male	51	44.3	19.6	45.1	35.3		
Female	64	55.7	25.0	50.0	25.0		
Age						2.72	.257
< 60 years	60	52.2	18.3	55.0	26.7		
≥ 60 years	55	47.8	27.3	40.0	32.7		
Education^f^						6.14	.046
Lower	68	59.6	23.5	**55.9**	**20.6**		
Higher	46	40.4	21.7	**37.0**	**41.3**		
BMI						3.41	.182
< 25	41	36.9	12.2	53.7	34.1		
≥ 25	70	63.1	27.1	44.3	28.6		
Cancer entity						4.54	.338
Breast	60	52.2	25.0	51.7	23.3		
Colon	14	12.2	28.6	50.0	21.4		
Prostate	41	35.7	17.1	41.5	41.5		
Metastases						7.62	.022
Yes	22	19.1	18.2	**72.7**	**9.1**		
No	93	80.9	23.7	**41.9**	**34.4**		
Comorbidity						6.49	.039
Yes	59	55.1	**30.5**	42.4	27.1		
No	48	44.9	**10.4**	58.3	31.3		
Side Effects^g^						9.25	.010
< 3	60	52.2	25.0	**35.0**	**40.0**		
≥ 3	55	47.8	20.0	**61.8**	**18.2**		
Health Status^g^						0.37	.832
< 5	41	38.7	17.1	53.7	29.3		
≥ 5	65	61.3	20.0	47.7	32.3		
Meeting PA guidelines^h^						1.81	.404
Yes	66	57.4	18.2	51.5	30.3		
No	49	42.6	28.6	42.9	28.6		

*PA* physical activity; values in bold indicate significant differences in the proportions of the two groups in the reported counseling behavior according to z-test with Bonferroni correction

^a^Indicated by 22.6% of participants overall

^b^At least once reported the counseling step *Ask* and/or *Assess* (indicated by 47.8% of participants)

^c^At least once reported the counseling step *Agree, Assist,* and/or *Arrange* (indicated by 29.6% of participants)

^d^Group differences were calculated using Chi Square tests

^e^Numbers may not sum up to total N due to missing values

^f^Lower = secondary education degree or lower; higher = graduation qualifying for university or higher

^g^Subgroups according to median value; side effects assessed at t1

^h^Yes = at least 150 min at least moderate physical activity per week according to accelerometer-data at baseline; no = less than 150 min at least moderate physical activity per week according to accelerometer-data at baseline

## Results

### Data availability and study flow

**Fig. 2:**
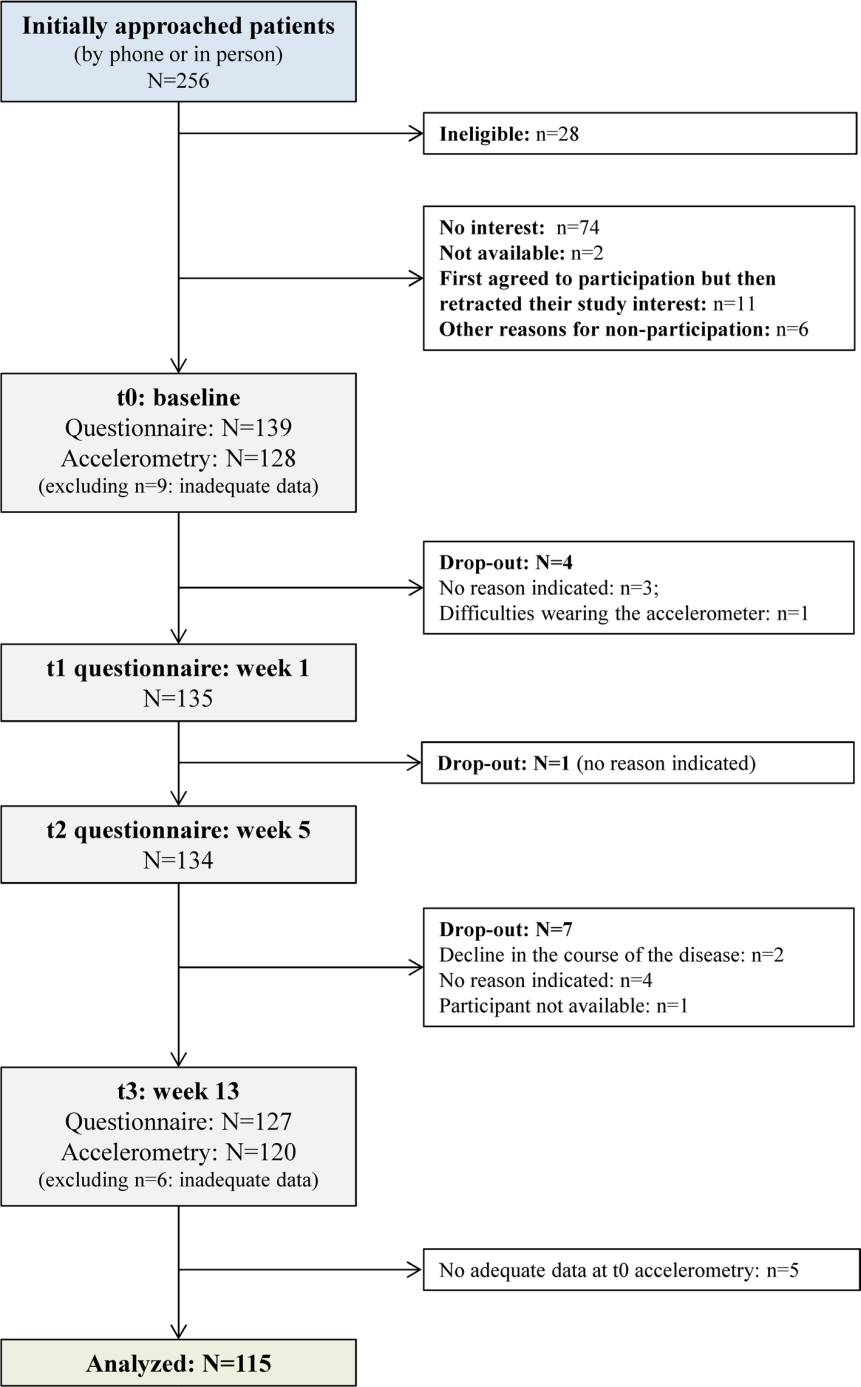
Flow diagram of the recruitment process and trough the phases of the study

Of the 256 individuals with cancer initially approached, 139 were eligible and agreed to study participation (54.3%; cf. Figure 2). Twelve participants dropped out before t3 (8.6%). A total of N = 115 participants provided valid accelerometer-data for both measurements and were included in the analyses. Thus, we did not reach our target of N = 180 participants despite intensive efforts and several recruitment routes. A sensitivity power analysis was conducted using G*Power (Faul et al., 2009) to determine the minimum detectable effect size with a sample size of N = 115. To approach the statistical power for structural equation models, we approximated the effect size that can be detected with a power of 80% (α = 0.05) for a linear multiple regression analysis with six predictors. For a sample of N = 115 participants, adequate power would be obtained to detect a true population effect of f^2^ = 0.125 (small to medium effect size).

Tests on differences between participants included (n = 115) and excluded (n = 24) for the final analyses suggested a lower self-reported health status of excluded participants, *Χ*^2^(1, N = 128) = 4.63, *p* = 0.031. All other comparisons regarding sociodemographic, health-related, and disease-related characteristics did not yield statistically significant differences.

### Descriptive statistics of the sample and TPB variables

Descriptive statistics of the final study population are displayed in Table 1. Participants had a mean (M) age of *M* = 58.0 (standard deviation (*SD*) = 11.5) and 55.7% were female. The median time since diagnosis at baseline was 2 months (89.6% had received the diagnosis no more than 4 months ago) and 19.1% were diagnosed with metastases. During the study period, n = 52 (45.2%) participants underwent chemotherapy, n = 30 (26.1%) radiotherapy, n = 16 (13.9%), immunotherapy, and n = 23 (20%) hormone therapy; n = 37 (32.2%) reported more than one therapy type. Thirty-four (29.6%) participants had surgery only, without subsequent treatment.

Descriptive statistics of TPB variables at t1 showed that the mean values for activity attitude (*M* = 4.12, *SD* = 0.70, range 1–5), self-efficacy (*M* = 5.50, *SD* = 1.80, range 1–7), and intention (*M* = 6.00, *SD* = 1.37, range 1–7) were in the upper range of the scale. Their standard deviations indicate, however, a substantial variance in the participants' data. Participants’ attitudes towards activity were significantly higher than towards rest (*M* = 2.53, *SD* = 0.68; *t*(114) = 14.15, *p* < 0.001), indicating that participants were more likely to endorse the benefits of PA during cancer treatment than the need for rest.

### Patterns of PA behavior

Overall, participants’ time spent in moderate-to-vigorous PA per week did not statistically change from the baseline (*M* = 215.60 minutes per week; *SD* = 170.28) to the t3 actigraphy measurement (*M* = 229.07 minutes per week; *SD* = 168.53). The change in participants' activity behavior from baseline to t3 can be attributed to the following subgroups: 50.4% met PA guidelines (i.e., at least 150 min moderate-to-vigorous PA per week) at both measurements (maintainers), 14% only at t3 (increasers), 8% only at baseline (decreasers), and 30.4% neither at baseline nor at t3 (consistently inactive).

Self-reported PA data show that participants engaged in moderate-to-vigorous PA for an average of 267.9 (*SD* = 330.3) minutes per week prior to their diagnosis and *M* = 140.4 (*SD* = 221.4) minutes in the week prior to baseline.

### Reported PA counseling in the course of the study

With regard to PA counseling behavior, 22.6% of participants reported that they never received any PA counseling by the physician before and during treatment, 47.8% reported receiving basic PA counseling at least once, and 29.6% reported that their attending physician provided an in-depth counseling at least once. Among those who indicated the “Advise” step, 80.5% reported that this was on the physician’s initiative at least once and 77.6% reported that this was on their own initiative at least once. Regarding the specialty of the attending physician who provided PA counseling, oncologists were indicated most frequently (47 mentions), followed by urologists (32), general practitioners (30), gynecologists (27), radiation therapists (19), surgeons (3), and gastroenterologists (1).

**Fig. 3:**
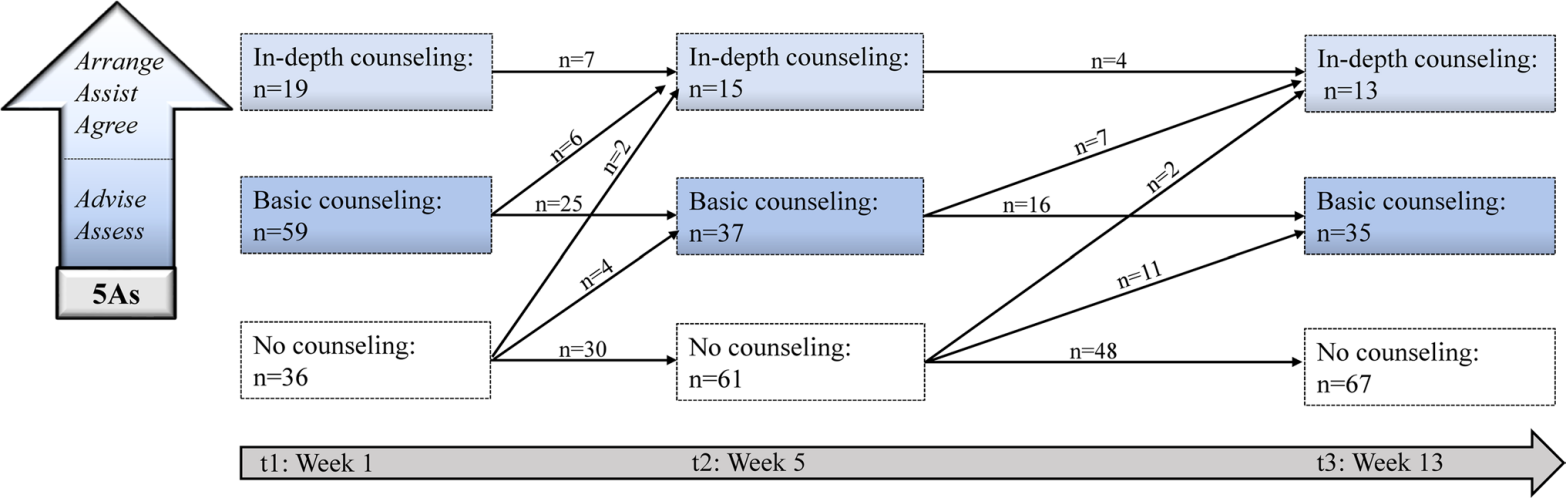
Patient-reported physical activity counseling provided by their attending physician during treatment/post-surgery. *Note:* The numbers indicated on the arrows refer to the number of participants who reported constant or more advanced physical activity counseling on the later assessment. For illustrative purposes, the number of participants who reported less advanced physical activity counseling on the later assessment is not indicated

Figure 3 provides details on the type of PA counseling by the attending physician during the course of the study: among participants who received PA counseling, the majority of participants reported that they had already received basic (n = 59, 51.3%) or in-depth counseling (n = 19, 16.5%) at t1. At the subsequent assessments (t2/t3), only a minority of participants reported receiving their first basic or in-depth PA counseling. For example, among those patients who did not receive any PA counseling at baseline (n = 36), only six reported that they had received basic or in-depth counseling during the next four weeks (at t2) and 13 eight weeks later (at t3).

### Differences in PA counseling as a function of sociodemographic and disease-related characteristics

It was investigated how sociodemographic and disease-related characteristics of the participants at baseline were associated with reported PA counseling by their attending physician during the study period (Table 1). Significant differences were found for education, metastases, comorbidity, and side effects. In detail, participants with higher education, no metastases and only a small number of side effects reported more often that they had received an in-depth counseling by their physician (education: Χ^2^ = 6.14, *p* = 0.046; metastases: Χ^2^ = 7.62, *p* = 0.022; side-effects: Χ^2^ = 9.25, *p* = 0.010). In this line, participants with comorbidities reported more frequently that they did not receive any PA counseling by their attending physician (30.5% compared to 10.4% without comorbidities; Χ^2^ = 6.49, *p* = 0.039). Regarding the different types of treatment (not shown in Table 1), there was a significant difference with respect to chemotherapy (Χ^2^ = 11.22, *p* = 0.004), with a lower number of those study participants who reported an in-depth counseling undergoing chemotherapy (13.5%) compared to those without chemotherapy during the study period (41.9%). Contrarily, those who had a surgery (without other therapy during the study period) reported more often in-depth PA counseling (47.1%) compared to those with other treatments (22.2%; Χ^2^ = 7.11, *p* = 0.029). There were no other significant differences regarding reported PA counseling based on treatment type.

### Longitudinal determinants of participants’ intention for PA (t2) and accelerometer-derived PA (t3)

**Fig. 4:**
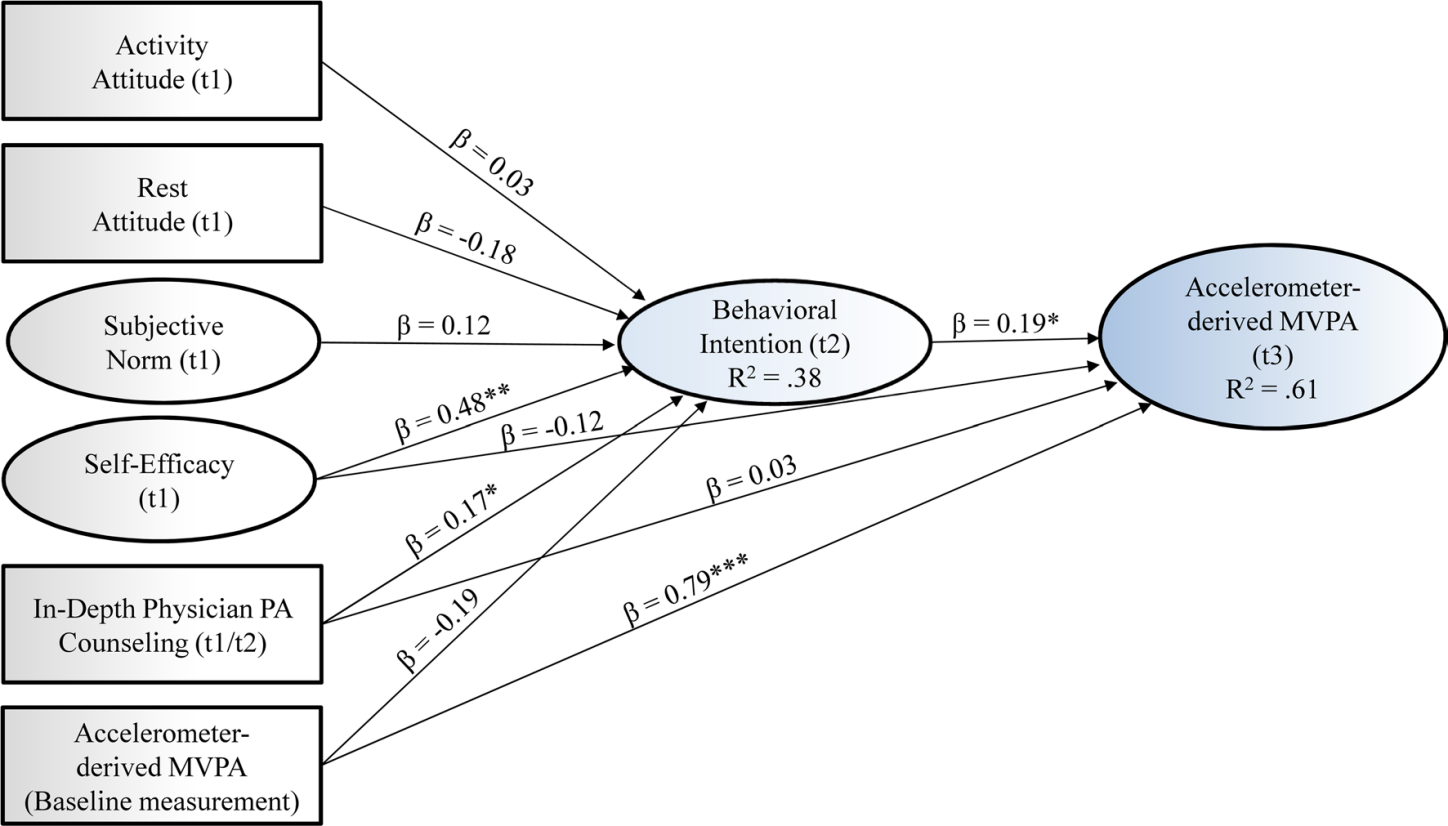
Structural equation model with Theory of Planned Behavior variables, physical activity counseling (i.e., in-depth counseling) and accelerometer-derived moderate-to-vigorous physical activity. *Note:* Standardized structural coefficients are shown. Measurement model and covariances between all variables are not displayed; MVPA = moderate-to-vigorous physical activity; PA = physical activity; R^2^ = explained variance; *p < .05; **p < .01; ***p < .001

Two structural equation models with TPB variables and PA counseling by the attending physician were calculated to indicate factors being associated with participants’ PA behavior at t3 (i.e., three months after baseline). One model included physician PA counseling in general without differentiation between basic and in-depth counseling, and one included in-depth counseling (cf. Online Resource 2 for estimates of both models). The structural equation model with in-depth PA counseling is presented in Fig. 4. Accelerometer-based moderate-to-vigorous PA at t3 was significantly predicted by moderate-to-vigorous PA at baseline (*b* = 0.73, *p* < 0.001, 95% CI [0.591, 0.869]) and the intention to regularly engage in at least moderate PA at t2 (i.e., 4 weeks after t1; *b* = 0.37, *p* = 0.031, 95% CI [0.033, 0.707]). Regarding explaining the PA intention at t2, in turn, self-efficacy (t1) was the only significant predictor based on the TPB (*b* = 0.17, *p* = 0.002, 95% CI [0.051; 0.289]). In addition, in-depth PA counseling explained PA intention above the TPB variables (*b* = 0.18, *p* = 0.045, 95% CI [0.002; 0.358]).

In contrast, within the model with PA counseling in general, physician PA counseling did not reveal a significant association with PA intention (cf. Online Resource 3). Both models showed a significant deviation between observed and expected results according to the Chi square test (both *p* < *0.0*5) but according to all other indices a good model fit (cf. Online Resource 4).

### Moderation of PA counseling in the effect of intention (t2) on accelerometer-derived PA (t3)

A moderation analysis was performed to test the moderating effect of physician PA counseling on the association between PA intention on PA behavior at t3. There was no significant interaction effect of physician PA counseling (i.e., no differentiation between basic counseling or in-depth counseling) in the effect of the intention at t2 on the moderate-to-vigorous PA eight weeks later (at t3; *p* = 0.561). Thus, the proposed moderation was not significant.

We exploratively tested if the proposed moderation would be significant for specific subgroups (cf. Online Resource 5 for conceptual model). Therefore, we calculated 3-way moderation models using all sociodemographic, disease-related variables and treatment types as second moderator. Health status turned out to be a significant second moderator (three-way interaction: β = 0.86, *p* = 0.002; cf. Online Resource 6). Thus, for the subgroup of people with high health status, intention was more likely transferred to higher PA behavior when they received a PA counseling by their attending physician.

## Discussion

This longitudinal observational study provided insights into how individuals with cancer experience PA counseling by their physician during their treatment, and into how this PA counseling and psycho-cognitive determinants based on the Theory of Planned Behavior (TPB) explain accelerometer-derived changes in PA. The majority of participants reported being asked or advised about PA by their physician (basic counseling) at least once during the study period, while only one in three reported receiving counseling beyond a PA recommendation (in-depth counseling). Participants in poorer health and those with lower education reported less frequently that they received in-depth PA counseling. Remarkably, only in-depth PA counseling, but not basic PA counseling, predicted the intention to engage in PA four weeks later. This intention for PA was additionally predicted by participants’ self-efficacy. Results further point to stable activity levels, with PA three months after baseline being most strongly predicted by PA at baseline. Physicians’ counseling (in-depth or basic PA counseling) neither predicted PA three months later nor moderated the effect of PA intention on actual PA levels two months later.

In this study, the majority of individuals with cancer received some kind of PA counseling by their physician during their (initial) treatment. PA counseling practices were assessed in detail according to the 5As framework (Estabrooks & Glasgow, 2006) and classified into *basic PA counseling* (assess and/or advise) and *in-depth PA counseling* (setting up goals, assist, and/or arrange follow-up monitoring). More participants reported that they received *basic PA counseling* (48%) than *in-depth PA counseling* (30%) at least once during the study. The percentage of participants who reported that they received a PA recommendation in this study was higher than in previous studies in cancer survivors (13–50%), which did not differentiate between the intensity of PA counseling (Eng et al., 2018; Fisher et al., 2015; Höh et al., 2018; Steele et al., 2021). In a previous cross-sectional study, a similar pattern was observed with respect to the intensity of PA counseling (basic PA counseling: 59%; in-depth PA counseling: 20%) (Haussmann et al., 2021). In line with this cross-sectional study, we found that nearly 1 in 4 participants received no PA counseling at all. Thus, despite numerous research findings on the benefits of PA, there remains a proportion of cancer survivors who do not receive (or at least do not remember) any PA counseling by their physician. For this subset of cancer survivors, structures should be created to guarantee counseling on PA at least once after cancer diagnosis, optimally through targeted PA screening at the beginning of therapy (as an example, cf. the Alberta Cancer Exercise PA screening form (Mina et al., 2018)).

Results of this study also indicate that cancer patients with more comorbidities are more likely to receive no PA counseling at all. In line with this result, cancer patients in poorer health (i.e., more side effects and diagnosis of metastases) and who were undergoing chemotherapy were advised less intensively about PA by their attending physician, i.e., they reported more basic but less in-depth PA counseling. In a previous cross-sectional study, a different physician counseling behavior was found for patients not meeting PA guidelines before diagnosis (more likely to receive no PA counseling at all) and patients who were currently under treatment (more likely to receive basic counseling). We are not aware of any study that has examined PA counseling longitudinally at the onset of cancer treatment, a time when patients’ health status often declines (Lewandowska et al., 2020; Yucel et al., 2014). Importantly, it is well-known that PA is associated with a reduced incidence of several comorbidities (Kang et al., 2018), and beneficial to reducing different side effects of cancer treatment (Campbell et al., 2019). Exemplarily, with regard to alleviating cancer-related fatigue, a meta-analysis of individual patient data indicated that patients with severe fatigue particularly benefited from exercise training (Buffart et al., 2018). In a previous cross-sectional study, cancer patients with metastases reported that they disproportionately increased their PA level from pre- to post-diagnosis when given in-depth PA counseling (Haussmann et al., 2021). Thus, providing fewer PA counseling to cancer patients in poor health seems counterintuitive, as these patients would strongly benefit from (in-depth) PA counseling. In our study, patients with a lower educational level were also less likely to report in-depth PA counseling. This finding is of high practical relevance as this group is at increased risk of not adhering to exercise guidelines anyway (Eng et al., 2018; Steindorf et al., 2019). Future research should focus more on how to reach individuals with a lower educational level, e.g., by analyzing which kind of information material is beneficial for which patient groups (Webb et al., 2019).

An important aim of this study was to identify determinants that can explain the intention for PA and the change in PA over the course of cancer treatment or post-surgery. The TPB set the framework for the investigated structural equation models, with patient-reported physician PA counseling as an additional variable. Overall, the structural equation models yielded a good model fit, confirming the applicability of the TPB for the longitudinal explanation of PA behavior (Hirschey et al., 2020). Within the model, participants’ intention for PA four weeks after baseline was predicted by patients’ self-efficacy for PA and physician PA counseling. With regard to PA counseling, however, this was only true for in-depth counseling, not PA counseling in general. The result may indicate that a simple recommendation may not be sufficient to achieve changes in patients’ intention to engage in PA. However, the effect was rather small (the lower limit of its CI was slightly above 0) and refers only to the intention after 4 weeks; future studies, optimally randomized controlled trials, should investigate whether the effect can be validated and sustained over a longer period.

Given their heavy workload, physicians treating oncological patients may struggle to provide this time-consuming PA counseling on a regular basis. Correspondingly, a lack of time was cited by healthcare professionals as one of the most major barriers to counsel cancer survivors on PA (Haussmann et al., 2018b; Nadler et al., 2017; Ramsey et al., 2022). For this reason, the ACSM (i.e., the Exercise is Medicine Initiative) recommends that oncologists refer cancer patients to outpatient rehabilitation specialists for further evaluation if they are unsure whether the patient can safely exercise (Schmitz et al., 2019). In this regard, a predefined pathway of PA promotion with clear procedures and responsibilities could facilitate physicians to integrate PA counseling. Physicians (or other qualified healthcare professionals) could be the starting point in this pathway by referring patients to exercise programs or, if not available, providing self-management tools (Mina et al., 2018). Recently published studies on intervention developments provide blueprints for how physicians and other healthcare professionals can be involved in promoting PA to oncology patients in a structured facilitating process (Kennedy et al., 2020; Rammant et al., 2021; Turner et al., 2021).

Self-efficacy to engage in sufficient PA was the only psycho-cognitive variable based on the TPB that showed a significant effect on patients’ intention for PA four weeks later. This is in line with the meta-analysis by Hirschey et al. (2020) in which self-efficacy turned out (together with attitudes) to be the strongest predictor of PA intention. Current studies on breast cancer patients during chemotherapy also showed a significant association between self-efficacy with PA levels (Auster-Gussman et al., 2022) and with better adherence to exercise programs (An et al., 2020).

Results of the structural path models further revealed that PA of study participants three months after baseline was most strongly predicted by PA at baseline. This finding indicates a high stability of PA behavior in our study participants, as also indicated by the high number of participants who either achieved (50%) or failed to achieve (30%) PA guidelines at each of the two accelerometer-based measurements. Previous research pointed to a decline in PA levels during cancer treatment (Huy et al., 2012; Kenkhuis et al., 2021) or post-surgery (Smith et al., 2019; Zhou et al., 2022), which suggests that the study population in this work was less likely to let the cancer therapy discourage them from their usual PA.

Intention to engage in sufficient PA was another significant predictor of PA behavior, although the regression coefficient in this study (β = 0.19) was not as high as in the meta-analysis of longitudinal studies by Hirschey et al. (2020) (β = 0.27). Contrary to our expectation, the association between participants’ intention for PA and PA levels eight weeks later was not moderated by physician counseling; thus, overall PA counseling did not contribute to turn PA intentions into action (nevertheless, exploratory analyses revealed that it did, though, for participants with a high health status). Although some studies point to an effect of a single PA recommendation on cancer survivors’ PA levels (e.g., Jones et al., 2004), there is also evidence that additional supporting material to an oncologist PA recommendation is beneficial to foster cancer patients’ PA (Park et al., 2015; Winters-Stone et al., 2018). Due to the small number of study participants that reported in-depth counseling between t2 and t3, we could not distinguish between basic and in-depth PA counseling. Therefore, we cannot exclude that specifically in-depth counseling may help to translate PA intention into actual PA. Behavior change techniques such as concrete goal setting and coping and action planning are effective to bridge the intention-behavior gap (Avishai et al., 2019; Sheeran & Webb, 2016), but there were contrary results for study populations with cancer patients (Finne et al., 2018). This suggests that individuals with cancer need closer guidance in using behavioral change techniques. Future studies may investigate whether physicians (or exercise professionals) can actually offer this required closer guidance by providing in-depth PA counseling.

The strength of this study is that it longitudinally analyzed a period in which PA and physician PA counseling are particularly important, i.e., in the (initial) treatment phase. In this context, physician counseling in the form of the 5As framework and psycho-cognitive variables were assessed in detail at several time points. In addition, PA was objectively measured twice using accelerometers, resulting in a high data quality that is not biased by over or underestimation of own PA in self-reports (Prince et al., 2008). Results of this study provide valuable insights into what PA counseling is needed from a physician to influence PA intentions of patients during cancer treatment. The development of future interventions to encourage physicians to improve their PA counseling to individuals with cancer may build on these findings.

However, the results of this study must be considered in light of some limitations. The study sample might be subject to a selection bias, meaning that individuals with a high affinity for PA in particular participated in the study. According to retrospective self-reports, our study participants were on average already very active before their cancer diagnosis. This would explain the high level of PA at both measurements, which far exceeds the results of previous studies that objectively measured PA in cancer survivors (McDonald et al., 2019; Smith et al., 2019; Thraen-Borowski et al., 2017). Interestingly, at baseline, the self-reported moderate-to-vigorous-PA during the last week (before they wore the accelerometer the first time) was considerably lower. This is in line with research showing that wearing accelerometers can be an impetus to increase PA with similar effects as PA interventions that include additional behavioral change techniques (Brickwood et al., 2019). Potentially, the wearing period of seven days (with the minimum criterion of four wearing days) in this study was set too short to compensate for this effect. High mean values were also found regarding the psycho-cognitive variables of the TPB. These indicate ceiling effects, which may have led to under-estimations of the effects on intention in particular. The sample size was relatively small for the SEM analyzed which increases the risk of false-negative results (type 2 error) or may have caused the magnitude of effects found to be overestimated. Therefore, results have to be interpreted with caution. To account for the uncertainty in the results, we provided confidence intervals that allow a more accurate assessment of the effects. Furthermore, we conducted a sensitivity analysis showing that the study had adequate power to detect small to medium effects. We decided not to include any control variables in the two calculated models to ensure parsimony. As a result, our N exceeds the rule of thumb of a minimum of a 10:1 ratio of sample size to parameters in SEM (Kline, 2010). On the other side, not including control variables—such as medical (e.g., diagnosis of metastases) or sociodemographic variables (e.g., education)—in the model may also have resulted in an over-estimation of the effects found (omitted variable bias). This would be the case if the omitted control variable was positively associated with another predictor and had a positive effect on intention (t2) or on moderate-to-vigorous PA (t3). Due to the small sample size, we also could not conduct subgroup comparisons (as originally intended with regard to different cancer entities). In explaining PA intention and PA behavior in our study using the TPB, it may also be that additional variables (e.g., health-related variables or illness perceptions) would have been significant predictors. We did not use a controlled but an observational study design. Therefore, there is the risk that self-reported information was subject to biases, such as recall errors or socially desirable response behavior. Time points of received PA counseling could only be specified to the time between questionnaires (or time since diagnosis) and not to an exact date. Moreover, advice on PA could have been given by persons (other healthcare professionals or friends/family) other than the treating physician, who, however, seems to be a central source for PA advice for individuals with cancer (Avancini et al., 2020).

In this longitudinal study, we identified PA behavior at baseline as the strongest predictor of moderate-to-vigorous PA behavior during cancer treatment. However, in-depth counseling of physicians appears to make a difference by prompting higher intentions to engage in PA. At the same time, further research is needed how physicians can help translate the intention for PA into sufficient activity levels. In-depth PA counseling that integrates individualized guidance as part of a structured pathway in clinical care, seems to be a promising way forward. Our study also revealed that patients in poorer health and patients with lower education are less likely to receive an in-depth PA counseling. Therefore, a focus should be placed on ensuring that patient subgroups with different sociodemographic and medical backgrounds receive the optimal supportive care regarding PA.

### Supplementary Information

Below is the link to the electronic supplementary material.

Supplementary file1 (PDF 82 kb)

Supplementary file2 (PDF 35 kb)

Supplementary file3 (PDF 117 kb)

Supplementary file4 (PDF 89 kb)

Supplementary file5 (PDF 122 kb)

Supplementary file6 (PDF 123 kb)

## Data Availability

The authors declare that they have full control of all primary data and that they agree to allow the journal to review our data.

## Code availability

The authors declare that they agree to allow the journal to review our program code.
