# Supplementary material for: Determinants of physical activity during cancer treatment: a longitudinal exploration of psycho-cognitive variables and physician counseling
Source: J Behav Med. 2023 Nov 28;47(4):566–80. doi: 10.1007/s10865-023-00458-y (PMC11291613; doi:10.1007/s10865-023-00458-y)
Supplement: Supplementary file 2 — Supplementary file2 (PDF 35 kb) [file 10865_2023_458_MOESM2_ESM.pdf]

Title: Determinants of physical activity during cancer treatment: A longitudinal exploration of psycho-cognitive variables and physician counseling

Journal Name: Journal of Behavioral Medicine

Authors: Alexander Haussmann, Nadine Ungar, Angeliki Tsiouris, Laura I. Schmidt, Jana Müller, Jost von Hardenberg, Joachim Wiskemann, Karen Steindorf, Monika Sieverding

Corresponding Author: Alexander Haussmann, German Cancer Research Center and National Center for Tumor Diseases Heidelberg, alexander.haussmann@nct-heidelberg.de

Online Resource 2. Unstandardized regression weights of the proposed models

**Paths in Model with Physical Activity Counseling in General**[a]	**Estimate**	**Standard Error**	**Critical Ratio**	**95% CI**	**p-value**
Activity Attitude → Intention	0.06	0.09	0.70	[-0.118; 0238]	.486
Rest Attitude → Intention	-0.14	0.09	-1.53	[-0.318; 0.038]	.127
Subjective Norm → Intention	0.04	0.03	1.43	[-0.019; 0.099]	.154
Self-Efficacy → Intention	0.18	0.06	3.11	[0.061; 0.299]	.002
Basic PA Counseling → Intention	-0.05	0.07	-0.69	[-0.189; 0.089]	.493
MVPA at t0 → Intention	-0.10	0.05	-1.92	[-0.199; 0.001]	.055
MVPA at t0 → MVPA at t3	0.73	0.07	10.41	[0.591; 0.869]	<.001
Self-Efficacy → MVPA at t3	-0.10	0.08	-1.23	[-0.259; 0.059]	.219
Basic PA Counseling → MVPA at t3	0.07	0.09	0.77	[-0.108; 0.248]	.439
Intention → MVPA at t3	0.42	0.18	2.33	[0.063; 0.777]	.020
**Paths in Model with In-depth Physical Activity Counseling**					
Activity Attitude → Intention	0.02	0.09	0.25	[-0.158; 0.198]	.801
Rest Attitude → Intention	-0.15	0.09	-1.71	[-0.328; 0.028]	.088
Subjective Norm → Intention	0.03	0.03	1.05	[-0.029; 0.089]	.296
Self-Efficacy → Intention	0.17	0.06	3.09	[0.051; 0.289]	.002
In-depth PA Counseling → Intention	0.18	0.09	2.00	[0.002; 0.358]	.045
MVPA at t0 → Intention	-0.09	0.05	-1.80	[-0.189; 0.009]	.072
MVPA at t0 → MVPA at t3	0.73	0.07	10.42	[0.591; 0.869]	<.001
Self-Efficacy → MVPA at t3	-0.08	0.08	-1.11	[-0.239; 0.079]	.269
Basic PA Counseling →MVPA t3	0.07	0.13	0.55	[-0.188; 0.328]	.584
Intention → MVPA at t3	0.37	0.17	2.15	[0.033; 0.707]	.031

[a] No differentiation between basic and in-depth physical activity counseling

Note: CI=confidence interval; MVPA = moderate-to-vigorous physical activity
