# Supplementary material for: Determinants of physical activity during cancer treatment: a longitudinal exploration of psycho-cognitive variables and physician counseling
Source: J Behav Med. 2023 Nov 28;47(4):566–80. doi: 10.1007/s10865-023-00458-y (PMC11291613; doi:10.1007/s10865-023-00458-y)
Supplement: Supplementary file 3 — Supplementary file3 (PDF 117 kb) [file 10865_2023_458_MOESM3_ESM.pdf]

Title: Determinants of physical activity during cancer treatment: A longitudinal exploration of psycho-cognitive variables and physician counseling

Journal Name: Journal of Behavioral Medicine

Authors: Alexander Haussmann, Nadine Ungar, Angeliki Tsiouris, Laura I. Schmidt, Jana Müller, Jost von Hardenberg, Joachim Wiskemann, Karen Steindorf, Monika Sieverding

Corresponding Author: Alexander Haussmann, German Cancer Research Center and National Center for Tumor Diseases Heidelberg, alexander.haussmann@nct-heidelberg.de

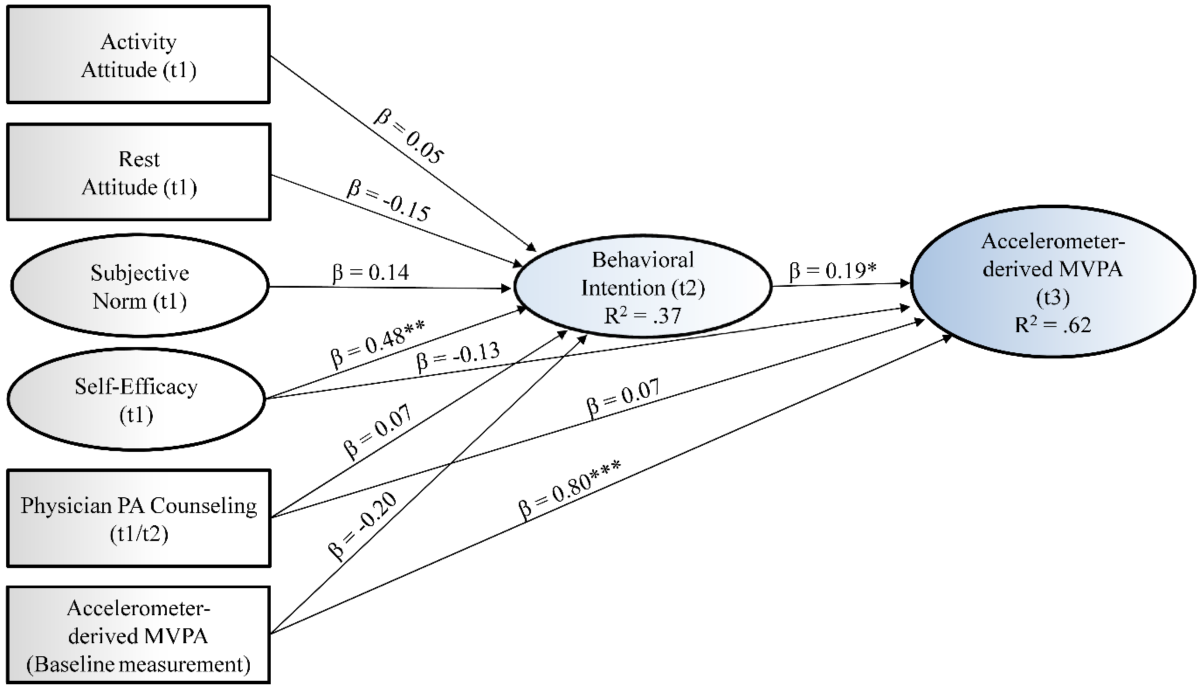

Online Resource 3. Structural equation model with Theory of Planned Behavior variables, physician physical activity counseling (i.e., basic or in-depth counseling) and accelerometer-derived moderate-to-vigorous physical activity.

Note: Standardized structural coefficients are shown. Measurement model and covariances between all variables are not displayed. MVPA=moderate-to-vigorous physical activity; PA=physical activity; R^2=explained variance; *p<.05; **p<.01; ***p<.001.
