# Supplementary material for: Determinants of physical activity during cancer treatment: a longitudinal exploration of psycho-cognitive variables and physician counseling
Source: J Behav Med. 2023 Nov 28;47(4):566–80. doi: 10.1007/s10865-023-00458-y (PMC11291613; doi:10.1007/s10865-023-00458-y)
Supplement: Supplementary file 4 — Supplementary file4 (PDF 89 kb) [file 10865_2023_458_MOESM4_ESM.pdf]

Title: Determinants of physical activity during cancer treatment: A longitudinal exploration of psycho-cognitive variables and physician counseling

Journal Name: Journal of Behavioral Medicine

Authors: Alexander Haussmann, Nadine Ungar, Angeliki Tsiouris, Laura I. Schmidt, Jana Müller, Jost von Hardenberg, Joachim Wiskemann, Karen Steindorf, Monika Sieverding

Corresponding Author: Alexander Haussmann, German Cancer Research Center and National Center for Tumor Diseases Heidelberg, alexander.haussmann@nct-heidelberg.de

Online Resource 4. Model fits of the proposed models

**Fit Index**	**Cut-off Value[a]**	**Model: Counseling in General[b]**	**Model: In-depth Counseling**
χ^2		57.86**	51.05*
CMIN/df	≤ 3.0	1.70	1.50
CFI	≥ 0.9	0.96	0.97
TLI	≥ 0.9	0.91	0.93
RMSEA	≤ 0.08	0.08	0.07

[a] Cut-off values for good model fit

[b] No differentiation between basic and in-depth physical activity counseling

Note: * = χ^2 test: $p < .05$; ** = χ^2 test: $p < .01$; CMIN/df = Ratio of Chi squared value and degrees of freedom; CFI = Comparative Fit Index; TLI = Tucker-Lewis Index; RMSEA = Root Mean Square Error of Approximation.
