# Supplementary material for: Determinants of physical activity during cancer treatment: a longitudinal exploration of psycho-cognitive variables and physician counseling
Source: J Behav Med. 2023 Nov 28;47(4):566–80. doi: 10.1007/s10865-023-00458-y (PMC11291613; doi:10.1007/s10865-023-00458-y)
Supplement: Supplementary file 5 — Supplementary file5 (PDF 122 kb) [file 10865_2023_458_MOESM5_ESM.pdf]

Title: Determinants of physical activity during cancer treatment: A longitudinal exploration of psycho-cognitive variables and physician counseling

Journal Name: Journal of Behavioral Medicine

Authors: Alexander Haussmann, Nadine Ungar, Angeliki Tsiouris, Laura I. Schmidt, Jana Müller, Jost von Hardenberg, Joachim Wiskemann, Karen Steindorf, Monika Sieverding

Corresponding Author: Alexander Haussmann, German Cancer Research Center and National Center for Tumor Diseases Heidelberg, alexander.haussmann@nct-heidelberg.de

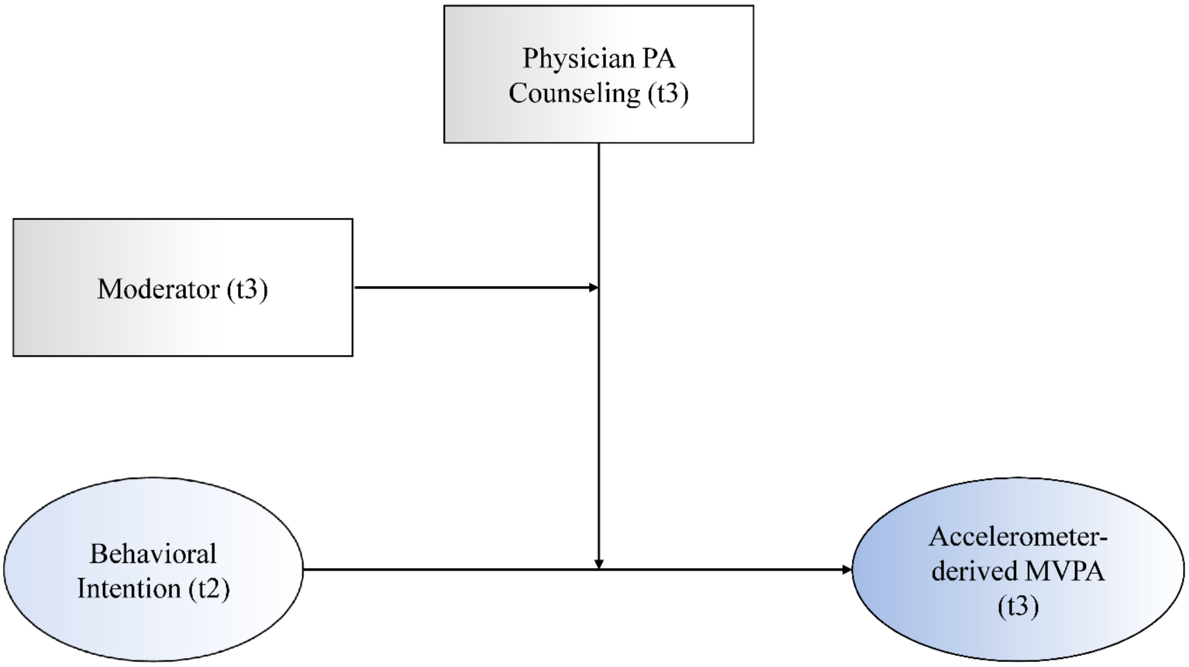

Online Resource 5. Conceptual research model with the three-way interaction of physician physical activity counseling (t3) and a moderator (i.e., sociodemographic or disease-related characteristic) (t3) regarding the effect of participants' intention for physical activity (t2) on their moderate-to-vigorous physical activity (t3).

Note: MVPA=moderate-to-vigorous physical activity; PA=physical activity.
