# Supplementary material for: Determinants of physical activity during cancer treatment: a longitudinal exploration of psycho-cognitive variables and physician counseling
Source: J Behav Med. 2023 Nov 28;47(4):566–80. doi: 10.1007/s10865-023-00458-y (PMC11291613; doi:10.1007/s10865-023-00458-y)
Supplement: Supplementary file 6 — Supplementary file6 (PDF 123 kb) [file 10865_2023_458_MOESM6_ESM.pdf]

Title: Determinants of physical activity during cancer treatment: A longitudinal exploration of psycho-cognitive variables and physician counseling

Journal Name: Journal of Behavioral Medicine

Authors: Alexander Haussmann, Nadine Ungar, Angeliki Tsiouris, Laura I. Schmidt, Jana Müller, Jost von Hardenberg, Joachim Wiskemann, Karen Steindorf, Monika Sieverding

Corresponding Author: Alexander Haussmann, German Cancer Research Center and National Center for Tumor Diseases Heidelberg, alexander.haussmann@nct-heidelberg.de

Online Resource 6. Moderated-moderation regression coefficients and confidence intervals for predicting moderate-to-vigorous physical activity per week (t3)

Interaction	β	SE	95% CI	p
Intention t2 x Physician Counseling[a] t3	0.10	0.30	[-0.49; 0.69]	.740
Intention t2 x Health Status[b] t3	-0.26	0.13	[-0.51; -0.02]	.038
Physician Counseling[a] t3 x Health Status[b] t3	-0.13	0.14	[-0.41; 0.16]	.395
Intention t2 x Physician Counseling[a] t3 x Health Status t3	0.86	0.27	[0.33; 1.39]	.002

Note: β=standardized regression coefficient; SE=standard error; CI=confidence interval; Analyses performed using the PROCESS macro for SPSS (Model 3; Hayes 2022). Model includes age, sex, BMI (t2), metastasis, current chemotherapy, sum of side effects (t3), self-efficacy (t1; mean), and moderate-to-vigorous physical activity (baseline measurement) as control variables; intention and moderate-to-vigorous physical activity per week were transformed using their natural logarithm.

[a] No=0; yes=1 (no differentiation between basic and in-depth physical activity counseling)

[b] Assessed via Short-Form-Health-Survey-12
